# Supplementary material for: Response of Rice with Overlapping Growth Stages to Water Stress by Assimilates Accumulation and Transport and Starch Synthesis of Superior and Inferior Grains
Source: Int J Mol Sci. 2022 Sep 22;23(19):11157. doi: 10.3390/ijms231911157 (PMC9569491; doi:10.3390/ijms231911157)
Supplement: Supplementary file 1 [file ijms-23-11157-s001.zip › Supplementary Figures.pdf]

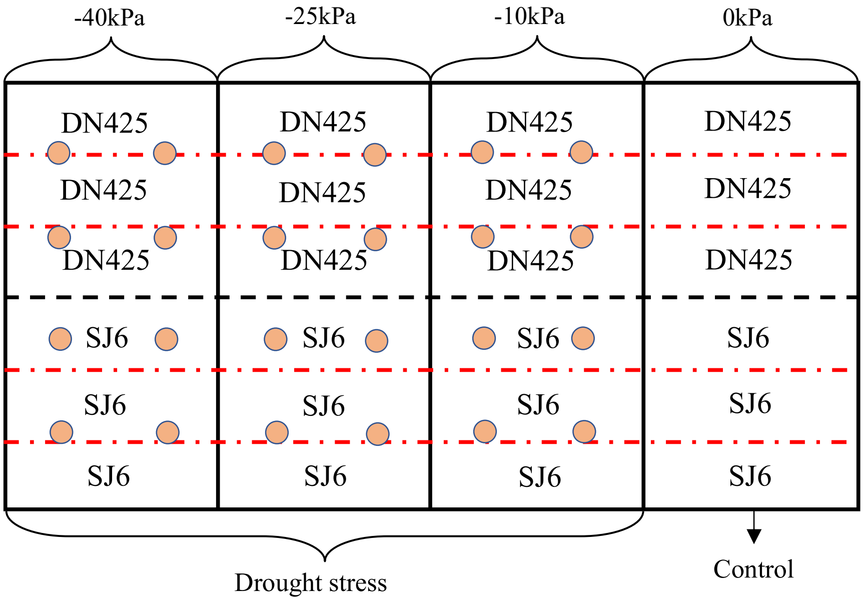

Figure S1. Field map of the experimental design.

Note: The red dotted line represents the separation between repetitions, the black dotted line represents the partition between varieties. The circle represents the position of the soil tensiometer.

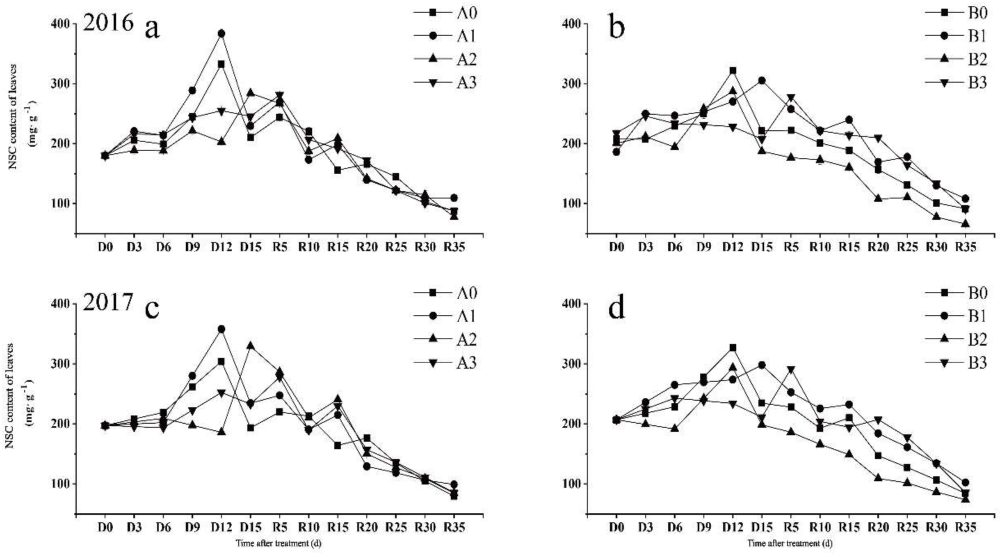

Figure S2. Effects of drought stress at the jointing–booting stage on NSC content of leaves in growth period overlapping rice in 2016 (**a**,**b**) and 2017 (**c**,**d**).

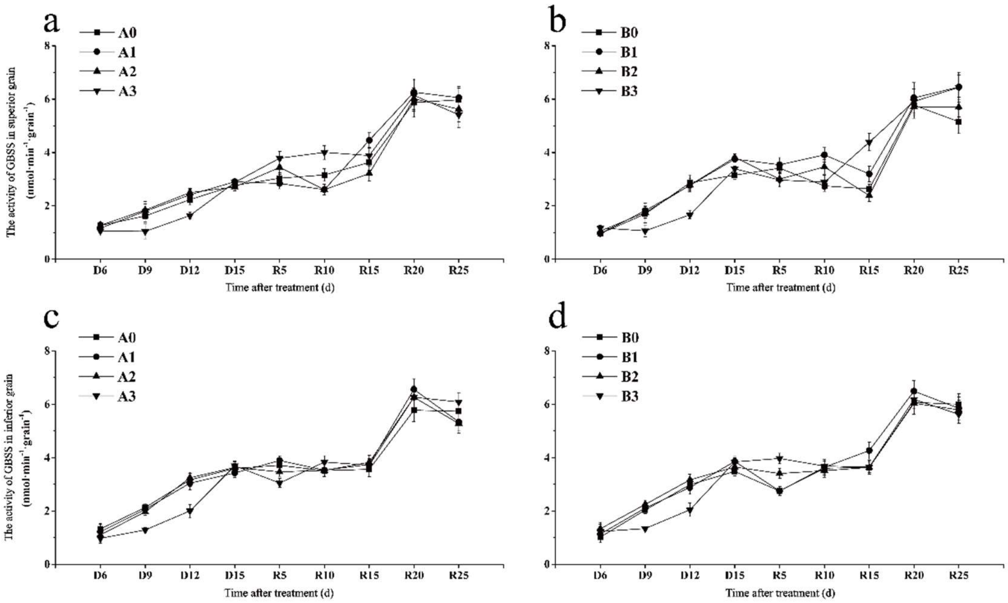

Figure S3. Effects of drought stress at the jointing–booting stage on GBSS activity of superior (**a**,**b**) and inferior (**c**,**d**) grains in growth period overlapping rice.

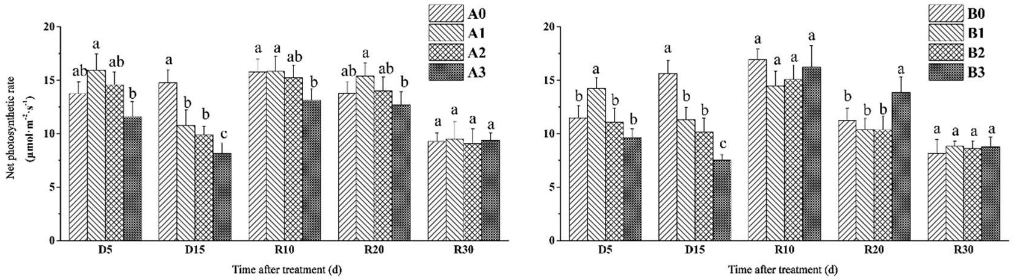

Figure S4. Effects of drought stress at the jointing–booting stage on net photosynthetic rate in growth period overlapping rice.

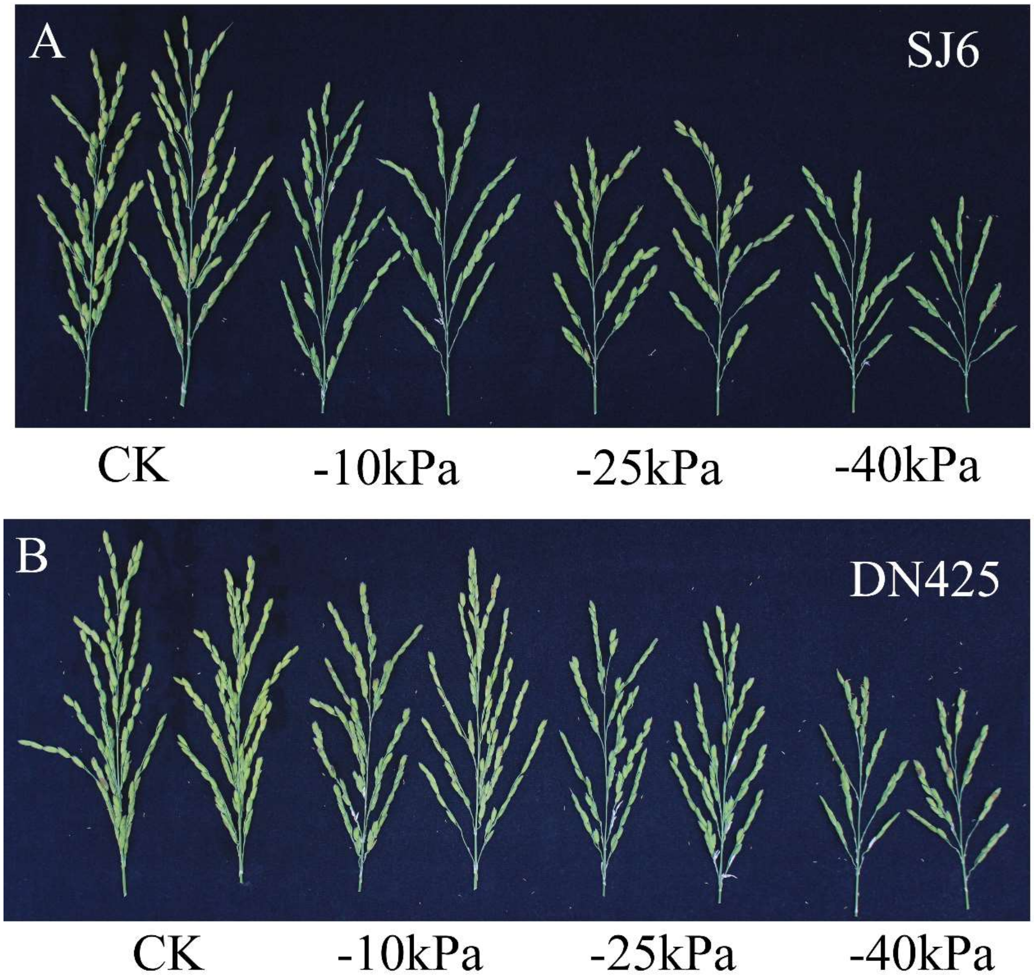

Figure S5. Effects of drought stress at the jointing–booting stage on panicle traits in growth period overlapping rice.
